# A Home-Based eHealth Intervention for an Older Adult Population With Food Insecurity: Feasibility and Acceptability Study

**DOI:** 10.2196/26871

**Published:** 2021-08-31

**Authors:** Luís Antunes Gomes, Maria João Gregório, Tatiana A Iakovleva, Rute Dinis de Sousa, John Bessant, Pedro Oliveira, Jaime C Branco, Helena Canhão, Ana Maria Rodrigues

**Affiliations:** 1 Comprehensive Health Research Centre NOVA Medical School Universidade NOVA de Lisboa Lisbon Portugal; 2 EpiDoC Unit, Centro de Estudos de Doenças Crónicas NOVA Medical School Universidade NOVA de Lisboa Lisbon Portugal; 3 Faculdade de Ciências da Nutrição e Alimentação da Universidade do Porto Porto Portugal; 4 Stavanger Business School Stavanger Norway; 5 University of Exeter Business School Exeter United Kingdom; 6 Copenhagen Business School Copenhagen Denmark; 7 NOVA School of Business and Economics Universidade NOVA de Lisboa Lisbon Portugal; 8 Serviço de Reumatologia do Hospital Egas Moniz - Centro Hospitalar Lisboa Ocidental (CHLO-EPE) Lisbon Portugal

**Keywords:** food insecurity, eHealth, television app, elderly people, vulnerable population, cognitive behavioral strategy, health innovation, multidisciplinary program

## Abstract

**Background:**

Food insecurity is a global public health challenge, affecting predominately the most vulnerable people in society, including older adults. For this population, eHealth interventions represent an opportunity for promoting healthy lifestyle habits, thus mitigating the consequences of food insecurity. However, before their widespread dissemination, it is essential to evaluate the feasibility and acceptability of these interventions among end users.

**Objective:**

This study aims to explore the feasibility and acceptability of a home-based eHealth intervention focused on improving dietary and physical activity through an interactive television (TV) app among older adults with food insecurity.

**Methods:**

A pilot noncontrolled quasi-experimental study was designed with baseline and 3-month follow-up assessments. Older adult participants with food insecurity were recruited from 17 primary health care centers in Portugal. A home-based intervention program using an interactive TV app aimed at promoting healthy lifestyle behaviors was implemented over 12 weeks. Primary outcomes were feasibility (self-reported use and interest in eHealth) and acceptability (affective attitude, burden, ethicality, perceived effectiveness, and self-efficacy), which were evaluated using a structured questionnaire with a 7-point Likert scale. Secondary outcomes were changes in food insecurity (Household Food Insecurity Scale), quality of life (European Quality of Life Questionnaire with five dimensions and three levels and Functional Assessment of Chronic Illness Therapy-Fatigue), physical function (Health Assessment Questionnaire, Elderly Mobility Scale, grip strength, and regularity of exercise), and nutritional status (adherence to the Mediterranean diet).

**Results:**

A sample of 31 older adult individuals with food insecurity was enrolled in the 12-week intervention program with no dropouts. A total of 10 participants self-reported low use of the TV app. After the intervention, participants were significantly more interested in using eHealth to improve food insecurity (baseline median 1.0, IQR 3.0; 3-month median 5.0, IQR 5.0; *P*=.01) and for other purposes (baseline median 1.0, IQR 2.0; 3-month median 6.0, IQR 2.0; *P*=.03). High levels of acceptability were found both before and after (median range 7.0-7.0, IQR 2.0-0.0 and 5.0-7.0, IQR 2.0-2.0, respectively) the intervention, with no significant changes for most constructs. Clinically, there was a reduction of 40% in food insecurity (*P*=.001), decreased fatigue (mean −3.82, SD 8.27; *P*=.02), and improved physical function (Health Assessment Questionnaire: mean −0.22, SD 0.38; *P*=.01; Elderly Mobility Scale: mean −1.50, SD 1.08; *P*=.01; regularity of exercise: baseline 10/31, 32%; 3 months 18/31, 58%; *P*=.02). No differences were found for the European Quality of Life Questionnaire with five dimensions and three levels, grip strength, or adherence to the Mediterranean diet.

**Conclusions:**

The home-based eHealth intervention was feasible and highly acceptable by participants, thus supporting a future full-scale trial. The intervention program not only reduced the proportion of older adults with food insecurity but also improved participants’ fatigue and physical function.

**International Registered Report Identifier (IRRID):**

RR2-10.2196/resprot.6626

## Introduction

### Background

Food security is an essential prerequisite for a population to be healthy, active, and well-nourished. According to the World Health Organization, food security exists “when all people, at all times, have physical, social and economic access to sufficient, safe and nutritious food that meets their dietary needs and food preferences for an active and healthy life” [1]. As a comprehensive concept, food security depends on four essential dimensions: physical availability of food, economic and physical access to food, food use and maximization of consumption, and stability at all times [1]. When one or more of these dimensions are compromised, people or households are assumed to have food insecurity.

The multidimensional and self-perceived nature of food insecurity makes quantification challenging. Among the various instruments, the Household Food Insecurity Scale developed by the United States Department of Agriculture (USDA) is the most widely used tool in epidemiological studies [2,3]. This instrument classifies the food insecurity status of an entire household while considering multiple dimensions using a simple, easy, and validated approach [3].

Food insecurity is a global public health problem. Despite mainly affecting low-income countries, increasing evidence suggests that food insecurity is also highly prevalent in high-income countries [4]. For example, in the United States, one of the richest nations in the world, a USDA report states that 10.5% of households were facing food insecurity in 2019 [2]. Although this represents a lower prevalence since the Great Recession of 2008, the economic downturn caused by the COVID-19 pandemic may trigger an unprecedented food insecurity crisis [5,6]. National estimates in March and April 2020 indicate that the prevalence of this problem has more than tripled to 38%, which is the highest level of food insecurity ever measured in the United States [7].

In Europe, particularly Portugal, a country still recovering from an economic crisis, the impact of the COVID-19 pandemic is uncertain. The current economic, political, and social instability in this country may have overwhelming consequences, especially for individuals who are physically, economically, and socially vulnerable, such as older adults. Indeed, the prevalence of food insecurity among older adults is remarkably high, with a study from our group reporting that 23% of older Portuguese people experienced food insecurity in 2015 and 2016, which is higher than the prevalence among adults [8,9].

The consequences of food insecurity among older adults present major challenges to the society. The aging of this population, which has a poor overall health status, physical comorbidities, limited family contact or assistance, and low income, can compromise the motivation of older adults to adopt health behaviors that are compatible with an active and healthy lifestyle, particularly those related to a healthy diet and physical activity. For this population, which has special nutritional needs, food insecurity is associated with a poor nutritional status, physical disability, muscle weakness, depression and anxiety symptoms, and lower health-related quality of life (HRQoL), which in turn increases vulnerability to other conditions, hospitalization, and death [9-14].

Developing strategies to mitigate the consequences of food insecurity among older adults is essential for increasing their quality of life and well-being. However, the unique characteristics of this population make it difficult to conduct an experimental study, leaving this particularly vulnerable and growing population with low access to health care innovation.

The promotion of healthy lifestyle habits and the resulting increase in well-being and quality of life among older adults could be addressed by eHealth interventions, which are defined as the use of information and communication technologies (ICTs) for health [15]. These interventions are low-cost, personalized approaches capable of increasing the autonomy of older adults and their access to quality health care [16-18]. Emerging evidence shows that eHealth interventions for older adults have delivered promising results in terms of improving nutritional status [19] and physical activity [20,21] and have positive clinical outcomes for specific conditions such as fragility fracture [22] and cardiovascular disease [23]. However, unique characteristics of the lifestyle of older adults should be acknowledged to overcome the associated barriers before the wider implementations of eHealth interventions [18,19,24].

Among the available ICTs, television (TV) is the device mostly used by older people [25]. As most older people’s houses have a TV, this device could present an opportunity to successfully design and implement eHealth interventions aimed at promoting healthy lifestyles with their daily life. By improving the nutritional status and physical activity levels, a TV home-based program might slow the decline in physical function and increase the well-being and quality of life among older adults experiencing food insecurity.

**Figure 1:**
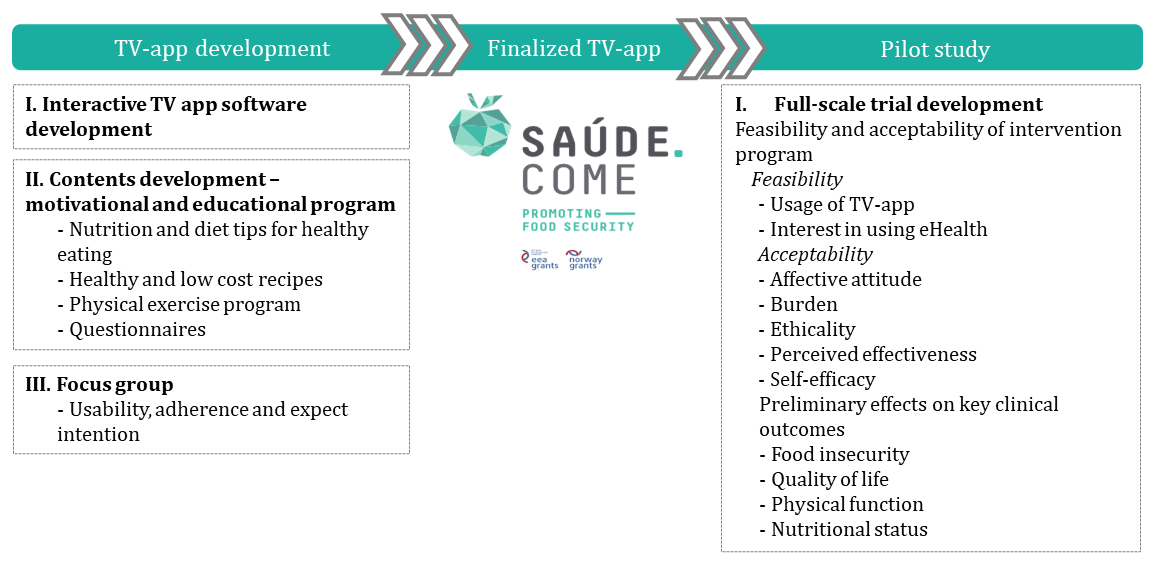
Conceptual framework of the program. TV: television.

To promote healthy lifestyles among older adults with food insecurity, we designed a home-based intervention program using an interactive TV app [26]. Following the new Medical Research Council’s guidance for developing and evaluating complex interventions [27], a multidisciplinary team of health professionals (ie, physicians, nurses, nutritionists, and physiotherapists), physical exercise experts, telecommunications companies, stakeholders, and end users collaboratively developed the conceptual framework of the program (Figure 1). In this development process, 11 older adult participants in a focus group reported high app usability, adherence intention, and the expected impact of the program on behavior modification [26]. All aspects of program conception considered ethical, social, economic, cultural, and environmental implications for all stakeholders, as suggested by the Responsible Innovation Framework [28].

### Objectives

After the conclusion of the TV app development process [26], the next step was to analyze the feasibility and acceptability of this intervention program in a pilot study. The primary aim of this pilot study is to test the feasibility and acceptability of a multidisciplinary 12-week home-based intervention program focused on improving dietary and physical activities through an interactive TV app among older adults with food insecurity. The secondary aim is to assess changes in the key clinical outcomes at the 3-month follow-up. The results of this pilot study are crucial for understanding end users’ interest and use of the TV app along with the suitability of the program to their needs.

## Methods

### Research Design

This pilot study used a noncontrolled quasi-experimental design with before and after measurements. Program feasibility, acceptability, and key clinical outcomes were evaluated at baseline and at the 3-month follow-up after the completion of the eHealth intervention. Older adult participants aged 60 years or above were recruited from 17 primary health care centers in the *Lisboa e Vale do Tejo* health region in Portugal.

### Participants

A convenience sample of older adults with food insecurity from 17 primary care centers in *Lisboa e Vale do Tejo* health region was used in this study. Individuals were included if they (1) were aged 60 years or above, (2) were classified as having food insecurity during recruitment [29], (3) were able and willing to give written informed consent and a phone number contact and comply with other requirements of the study protocol, (4) were a Portuguese speaker or able to understand Portuguese, (5) were noninstitutionalized and living in a private household in Portugal, (6) had electricity at home, and (7) had cable TV with a box at home. Individuals were excluded if they (1) were unable to comply with the study protocol (eg, hearing or visual loss and cognitive impairment), (2) had an absolute contraindication to exercise, (3) had cable TV operated by a company other than MEO, Vodafone, or NOS, and (4) had a household member involved in the study. Participants were recruited between November 2015 and May 2016. During the recruitment phase, all older adults who attended health services in the selected primary health care centers were invited to complete an initial screening questionnaire, which included a food insecurity scale [29]. Participants who agreed to complete the questionnaire were asked to provide their consent for further contact. All participants who identified as having food insecurity were invited to participate in this study. In case of acceptance, the first baseline assessment appointment was scheduled. This occurred during an appointment in a primary health care setting with a multidisciplinary team that included a physician, nurse, nutritionist, and physiotherapist. The physician asked for informed consent and checked the eligibility criteria.

### Intervention

A detailed description of the intervention can be found in a previous study [26], which briefly comprised a 12-week home-based intervention focused on the innovative use of an interactive TV app. The design of the intervention program was based on a transtheoretical model of behavior change [30] and aimed to promote lifestyle behavior changes among older individuals with food insecurity by (1) providing education on the importance of a healthy diet and physical activity among older adults, (2) demonstrating that low household income is not a barrier to healthy lifestyle habits and that it is possible to have a healthy diet and practice physical activity at low cost, and (3) providing motivation for adopting a healthy diet and physical activity habits to reduce noncommunicable diseases. The pillars of program content were divided into nutrition and diet tips for healthy eating, low-cost healthy recipes, and physical exercise programs. Different contents were specifically structured based on the thematic weeks. The themes for each week and the respective content of each video were developed by considering the different types of food most frequently used in daily life routine (eg, vegetables, water, and milk). Nutrition and diet tips were based on the explanations of the benefits and harms of eating certain foods, especially regarding their relationship with risk factors for noncommunicable diseases and nutritional requirements for older adults [31-34]. Low-cost healthy recipes were specifically designed for our program using a popular Portuguese TV chef and a nutritionist. Finally, the physical activity program was developed by physical exercise experts with the specific objective of promoting the practice of physical activity at home for at least 30 minutes thrice a week [35].

All program content was disseminated on a dedicated TV channel via the interactive TV app and delivered on a scheduled basis on specific days of the week for developing a program access routine. To increase the participants’ motivation, interactive TV reminders, including tips about healthy lifestyle habits, were sent on a weekly basis. In addition, participants had at their disposal the teams’ telephone numbers and options to contact them whenever necessary. The research team also made frequent contact with the participants to increase their adherence and overcome difficulties. The TV app software included the delivery of small questionnaires aimed at assessing participants’ compliance and learning during the intervention program. These questionnaires were delivered on a weekly basis and could be answered using TV remote control buttons. Any participant who opened the TV app fewer than 2 times during the intervention period was excluded from the data analysis.

### Outcomes

#### Overview

The primary outcomes were feasibility and acceptability of the intervention program. Secondary outcomes were changes in food insecurity, quality of life, physical function, and nutritional status after the intervention.

Data were collected using a structured questionnaire administered at two time points: before the intervention at study enrollment (baseline) and 3 months after the intervention (follow-up). The baseline assessment was performed by a multidisciplinary team (ie, physicians, nurses, nutritionists, and physiotherapists) at the primary care centers involved in the study using a computer-assisted personal interview system. At the 3-month follow-up, the assessment was performed by telephone using a computer-assisted personal interview system by a team of trained research assistants.

#### Baseline Characteristics

During baseline assessment, information was collected regarding sociodemographic (ie, gender, age, years of education, and marital status), socioeconomic (ie, employment status, household composition, household monthly income, and income perception), clinical (ie, food insecurity, BMI [kg/m^2^], anxiety and depression symptoms measured using the Hospital Anxiety and Depression Scale [36], and self-reported noncommunicable chronic diseases), and lifestyle characteristics (ie, alcohol intake profile, smoking habits, PREDIMED [Prevención con Dieta Mediterránea] score, perceptions of the importance and difficulty of healthy eating, frequency of watching TV, frequency of computer, videogame, tablet use, and frequency of internet use).

#### Feasibility and Acceptability of the Intervention Program

The measures of feasibility of the intervention program were (1) self-reported use of the TV app evaluated at 3-month follow-up and (2) interest in the use of eHealth evaluated with a structured questionnaire administered pre- (baseline) and postintervention (3-month follow-up). This questionnaire was also used to evaluate the acceptability of the intervention program. The acceptability evaluation was based on the theoretical framework of acceptability and included the following component constructs: affective attitude (“how an individual feels about the intervention”), burden (“perceived amount of effort required to participate in the intervention”), ethicality (“extent to which the intervention has good fit with an individuals’ value system”), perceived effectiveness (“extent to which the intervention is perceived as likely to achieve its purpose”), and self-efficacy (“individuals’ confidence that they can perform the behaviors required to participate in the intervention”) [37]. The structured questionnaire included 16 questions rated on a 7-point Likert scale with scores ranging from 1 (strongly negative/completely disagree) to 7 (strongly positive/completely agree). To understand participants’ perceptions in greater detail, the questionnaire included 3 additional questions using an open question approach to evaluate the most liked and disliked content of the intervention program (affective attitude construct) and the greatest difficulties in using the TV app (burden construct). Participants who initially self-reported low use of the TV app were not included in the evaluation of the interest construct of feasibility or the acceptability of the intervention program, as their experience with the TV app was limited. The reasons for the low use of the TV app were assessed using an open question approach.

#### Food Insecurity, Quality of Life, Physical Function, and Nutritional Status

Secondary outcomes were changes in food insecurity, quality of life, physical function, and nutritional status after the intervention. Food insecurity was measured using the Household Food Insecurity Scale, a scale adapted and validated for the Portuguese population from the USDA Household Food Security Survey Module [29]. The tool is applied at the individual level and collects data on food insecurity status for the whole household. Using a score ranging from 0 to 14, households were classified into different categories of food insecurity: food security (score of 0), low food insecurity (score between 1 and 5 for households with children and between 1 and 3 for households without children), moderate food insecurity (score between 6 and 9 for households with children and between 4 and 5 for households without children), and severe food insecurity (score between 10 and 14 for households with children and between 6 and 8 for households without children) [29].

Quality of life was evaluated using two different measures. The European Quality of Life Questionnaire with five dimensions and three levels (EQ-5D-3L) was used to measure HRQoL [38,39]. A higher EQ-5D-3L score corresponded to a higher quality of life. The Functional Assessment of Chronic Illness Therapy-Fatigue (FACIT-F) was used to evaluate fatigue in patients with chronic diseases [40]. This score ranges from 0 to 52, with higher scores representing less fatigue.

The physical function was evaluated using several instruments. The functional ability of the individuals was measured using the Health Assessment Questionnaire (HAQ) [41,42]. The final HAQ score ranges from 0 to 3 points, with higher scores corresponding to a lower functional ability. The Elderly Mobility Scale (EMS) was used to evaluate the performance and mobility of participants and has a score ranging from 0 to 20 [43,44], with higher scores indicating a greater degree of independence or mobility. A dynamometer was used to measure hand grip strength (lbs). Finally, data concerning exercise regularity (yes or no) and frequency (0, 1-2, 2-4, or ≥5 exercise sessions per week for a duration of at least 45 minutes) were recorded.

Nutritional status was classified based on the PREDIMED questionnaire, a 14-item questionnaire assessing adherence to the Mediterranean diet (MD) [8]. A score of ≥10 points indicated a high adherence to MD, and a score <10 points indicated a low adherence to MD [45].

### Statistical Analysis

The results were analyzed using SPSS Statistics (version 26.0, IBM Corp). Continuous variables are reported as mean and SD. Categorical variables are reported as frequencies or proportions. Changes in the feasibility and acceptability of the intervention program between baseline and 3-month follow-up are reported as median and IQR. The Wilcoxon signed-rank test or Sign test was used to assess differences after the intervention regardless of whether the data were symmetric. Differences in food insecurity, quality of life, physical function, and nutritional status between baseline and 3-month follow-up were assessed depending on the type of variable (continuous or ordinal), normal distribution, and data symmetry. The food insecurity results are summarized as frequencies and proportions in a contingency table. The Sign test was used to assess the differences between pre- and postintervention, as all participants had food insecurity at baseline. EQ-5D-3L, FACIT-F, HAQ, EMS, and hand grip strength data are reported as mean and SD, and differences at 3-month follow-up were evaluated using a 2-tailed paired *t* test, Wilcoxon signed-rank test, or Sign test. Regularity of exercise (yes or no) and PREDIMED (low or high adherence to MD) results were analyzed using frequencies and proportions in a contingency table, and differences were evaluated using McNemar tests for paired binary data. A significance level of 5% was considered as statistically significant.

### Ethical Issues

The study was performed in accordance with the principles established by the Declaration of Helsinki and was reviewed and approved by the NOVA Medical School Ethics Committee, the National Committee for Data Protection (*Comissão Nacional de Proteção de Dados*), and the Ethical Committee of the Regional Health Authority of *Lisboa e Vale do Tejo*.

## Results

### Baseline Characteristics

**Figure 2:**
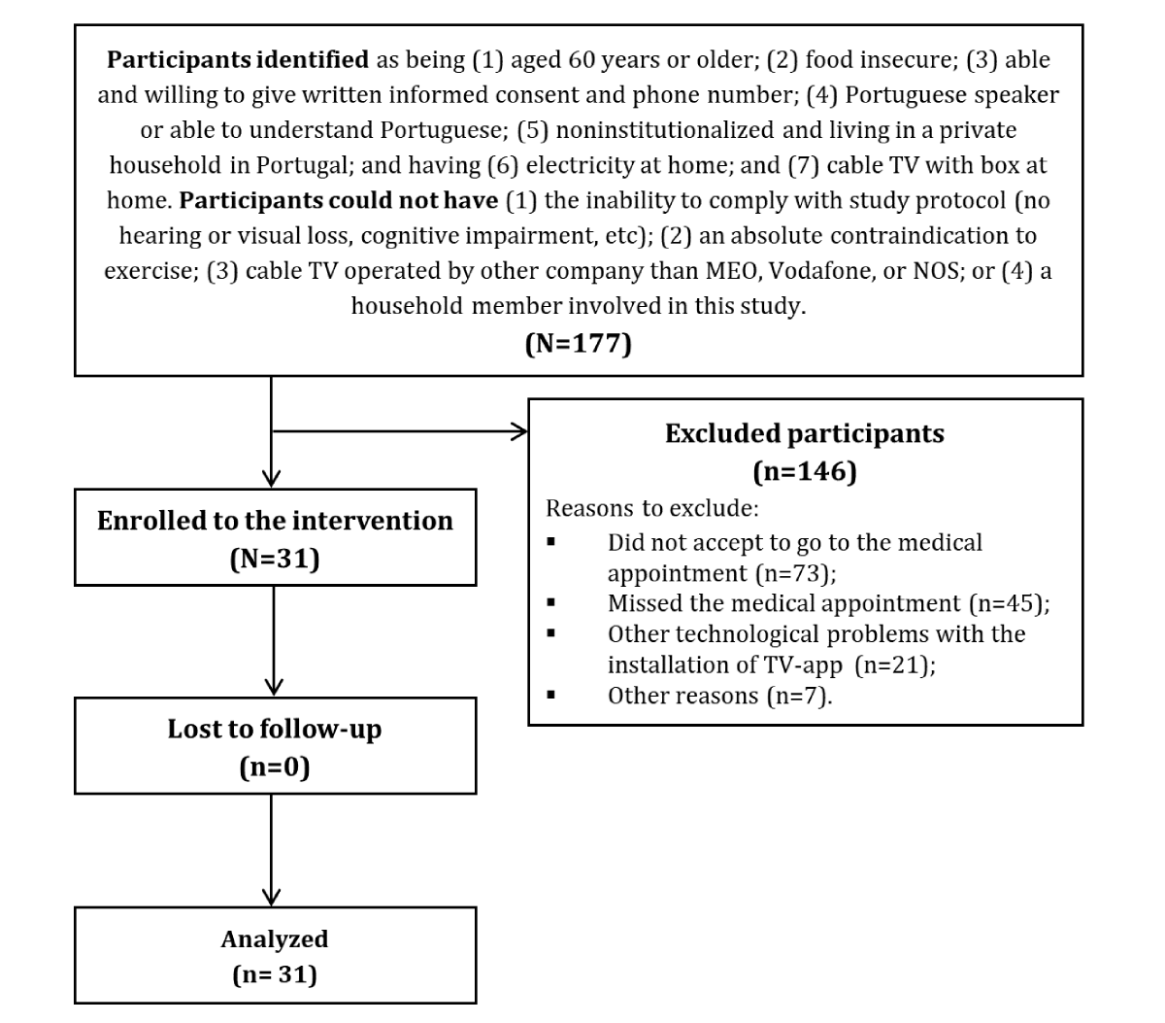
Participant recruitment and retention flowchart. TV: television.

During the 7-month recruitment period, 1857 individuals were screened at primary health care centers, 628 of whom were classified as having food insecurity. Of these, 177 older adult participants were identified as potentially eligible for the study; however, most did not agree to participate (73/177, 41.2%) or missed the medical appointment (45/177, 25.4%). Several participants (21/177, 11.9%) were excluded because of technological incompatibilities with the interactive TV app installation or software (Figure 2). Table 1 presents the participants’ baseline sociodemographic, socioeconomic, clinical, and lifestyle characteristics (n=31). The participants were predominantly female (21/31, 68%) with a mean age of 71.9 (SD 6.6) years and a low education profile (10/23, 43%). Most participants were retired (24/30, 80%), lived in a household with 3 or more people (9/23, 39%), and had a very low monthly income (13/23, 57%). Clinically, 50% (11/22) of the participants were obese, and most had chronic noncommunicable diseases. Regarding lifestyle, only 32% (10/31) were physically active and 90% (19/21) reported low adherence to MD. All participants acknowledged the importance of healthy eating (22/22, 100%), despite recognizing the associated difficulties (10/22, 45%). Finally, with a predominant watching frequency of 2-3 hours per day (13/31, 42%), TV was the predominantly used ICTs by participants. No participants were excluded from the data analysis at the 3-month follow-up.

**Table 1:** Baseline sociodemographic, socioeconomic, clinical, and lifestyle characteristics of participants (N=31)^a^.

Characteristics				Values
**Sociodemographic**				
	**Gender (n=31), n (%)**			
		Female	21 (68)	
	Age (years; n=31), mean (SD)			71.9 (6.6)
	**Age group (years; n=31), n (%)**			
		60-69	15 (48)	
		70-79	10 (32)	
		≥80	6 (19)	
	**Years of education (n=23), n (%)**			
		0-4	10 (43)	
		5-9	6 (26)	
		≥10	7 (30)	
	**Marital status (n=24), n (%)**			
		Single	3 (13)	
		Married	13 (54)	
		Divorced	6 (25)	
		Widow or widower	2 (8)	
**Socioeconomic, n (%)**				
	**Employment status (n=30)**			
		Employed	1 (3)	
		Unemployed	5 (17)	
		Retired	24 (80)	
	**Household composition (n=23)**			
		1 person	8 (35)	
		2 people	6 (26)	
		≥3 people	9 (39)	
	**Household monthly income (€; US $; n=23)**			
		≤750 (880.63)	13 (57)	
		751-1000 (881.80-1174.17)	5 (22)	
		>1000 (1174.17)	5 (22)	
	**Income perception (n=23)**			
		I live comfortably with my current income	1 (4)	
		I can live with my current income	7 (30)	
		It is difficult to live with my current income	7 (30)	
		It is very difficult to live with my current income	8 (35)	
**Clinical, n (%)**				
	**Food insecurity (n=31)**			
		Food security	0 (0)	
		Food insecurity	31 (100)	
		Low food insecurity	27 (87)	
		Moderate food insecurity	3 (10)	
		Severe food insecurity	1 (3)	
	**BMI (kg/m^2^; n=22)**			
		Underweight	0 (0)	
		Normal weight	3 (14)	
		Overweight	8 (36)	
		Obesity	11 (50)	
	**Anxiety and depression symptoms (n=23)**			
		Anxiety symptoms (HADS^b^ score ≥11)	7 (30)	
		Depression symptoms (HADS score ≥11)	3 (13)	
	**Noncommunicable chronic diseases (self-reported; n=31)**			
		Rheumatic disease	20 (65)	
		High blood pressure	17 (55)	
		Diabetes	7 (23)	
		High cholesterol	12 (39)	
		Pulmonary disease	2 (6)	
		Cardiac disease	5 (16)	
		Gastrointestinal disease	3 (10)	
		Neurologic disease	4 (13)	
		Neoplastic disease	3 (10)	
		Thyroid or parathyroid disease	5 (16)	
		Other diseases (eg, ophthalmic, dermatologic, or auditory)	3 (10)	
**Lifestyle habits, n (%)**				
	**Physical activity (n=31)**			
		Regular	10 (32)	
	**Alcohol intake profile (n=23)**			
		Daily	2 (9)	
		Occasionally	12 (52)	
		Never	9 (39)	
	**Smoking habits (n=31)**			
		Current smoker	4 (13)	
		Past smoker	9 (29)	
		Never	18 (58)	
	**PREDIMED^c^ (n=21)**			
		Low adherence to MD^d^	19 (90)	
		High adherence to MD	2 (10)	
	**Perceptions about the importance of healthy eating (n=22)**			
		Without or with little importance	0 (0)	
		Neither important nor unimportant	0 (0)	
		Important or very important	22 (100)	
	**Perceptions about the difficulty of healthy eating (n=22)**			
		Difficult or very difficult	10 (45)	
		Neither difficult nor easy	3 (14)	
		Easy or very easy	9 (41)	
	**Frequency of watching TV^e^ (n=31)**			
		Does not watch	1 (3)	
		≤1 hours/day	6 (19)	
		2-3 hours/day	13 (42)	
		≥4 hours/day	11 (35)	
	**Frequency of computer, videogame, or tablet use (n=31)**			
		Does not use	16 (52)	
		≤1 hours/day	6 (19)	
		2-3 hours/day	7 (23)	
		≥4 hours/day	2 (6)	
	**Frequency of internet use (n=31)**			
		Does not use	16 (52)	
		≤1 hours/day	8 (26)	
		2-3 hours/day	6 (19)	
		≥4 hours/day	1 (3)	

^a^Sample size is not constant due to missing data.

^b^HADS: Hospital Anxiety and Depression Scale.

^c^PREDIMED: Prevención con Dieta Mediterránea.

^d^MD: Mediterranean diet.

^e^TV: television.

### Feasibility and Acceptability of the Intervention Program

After the intervention program, some participants (n=10) self-reported low use of the TV app, and thus did not provide answers to the feasibility construct “interest in using eHealth” or acceptability questions. The reasons for the low use of the TV app were “lack of interest” (n=2), “lack of time” (n=2), “much time away from home during the intervention period” (n=2), “complications with the software” (n=1), or “health reasons” (n=1). Two participants did not provide a reason. Despite these results, all participants opened the TV app two or more times, and none were excluded from the data analysis of clinical outcome.

Regarding participants’ interest in using eHealth, a considerable increase was observed both for the treatment of food insecurity and other purposes.

Acceptability constructs showed very high scores both at baseline and at the 3-month follow-up, with no significant changes for most items (Table 2). However, “interest in using the TV app in daily life,” “attractiveness of using the TV app in daily life,” “advantages of using the TV app are/were higher than disadvantages,” and “ability of the TV app to solve challenges/problems in daily life” were significantly lower after the intervention program. The most liked contents were exercise (12/20, 60%), cooking (12/20, 60%), and nutrition (10/20, 50%) whereas the most dislike were exercise (5/20, 25%) and questionnaires (2/20, 10%). The main difficulties on using the TV app were “difficult in the access or manage the app” (6/20, 30%) and “lack of time” (4/20, 20%).

**Table 2:** Acceptability and feasibility of the television app intervention program (n=21)^a^.

Characteristics					Baseline^b^, median (IQR)			3-month follow-up^b^, median (IQR)			Difference^b^, median (IQR)			*P* value
**Feasibility**														
	Interest in using eHealth for treatment of food insecurity (n=20)			1.0 (3.0)			5.0 (5.0)			3.0 (5.0)			.01^c^	
	Interest in using eHealth for other purposes (n=20)			1.0 (2.0)			6.0 (2.0)			3.0 (4.0)			.03^c^	
**Acceptability**														
	**Affective attitude**													
		Desire to use TV^d^ app in daily life (n=19)	7.0 (1.0)			6.0 (2.0)			0.0 (2.0)			.06		
		Interest in using TV app in daily life (n=20)	7.0 (1.0)			6.0 (2.0)			0.0 (2.0)			.02^c^		
		Attractiveness of using the TV app in daily life (n=19)	7.0 (0.0)			6.0 (3.0)			0.0 (1.0)			.04^c^		
		Advantages of using the TV app are or were higher than disadvantages (n=19)	7.0 (0.0)			6.0 (3.0)			−1.0 (3.0)			.01^c^		
	**Burden**													
		Ease of using the TV app (n=21)	7.0 (1.0)			6.0 (3.0)			−1.0 (1.0)			.09		
	**Ethicality**													
		Ethical appropriateness of using digital technologies for treatment (n=20)	7.0 (0.0)			6.0 (1.0)			−1.0 (1.0)			.08		
		Acceptability of not including person-to-person interaction in medical treatment (n=20)	7.0 (2.0)			6.0 (2.0)			−1.0 (2.0)			.30		
		Suitability of using digital products to solve health problems (n=18)	7.0 (2.0)			5.0 (2.0)			−2.0 (3.0)			.08		
		Generally in favor of using technologies to treat people (n=20)	7.0 (0.0)			6.0 (2.0)			0.0 (2.0)			.14		
	**Perceived effectiveness**													
		Utility of TV app (n=19)	7.0 (0.0)			6.0 (2.0)			0.0 (2.0)			.10		
		Ability of TV app to solve challenges or problems in daily life (n=16)	7.0 (0.0)			5.0 (1.0)			−2.0 (3.0)			.002^c^		
	**Self-efficacy**													
		Motivation to use digital products (n=20)	7.0 (1.0)			7.0 (2.0)			0.0 (0.75)			.17		
		Control over TV app (n=20)	7.0 (1.0)			7.0 (2.0)			0.0 (1.5)			.47		
		Existence of circumstances beyond control that prevent use of TV app (n=20)	7.0 (1.0)			6.5 (4.0)			0.0 (2.5)			.05		

^a^Sample size is not constant due to missing data.

^b^Items rated on a 7-point Likert scale from 1 (strongly negative/completely disagree) to 7 (strongly positive/completely agree).

^c^*P*<.05.

^d^TV: television.

### Food Insecurity, Quality of Life, Physical Function, and Nutritional Status

Table 3 presents the changes in food insecurity, quality of life, physical function, and nutritional status. The intervention program significantly reduced food insecurity status and severity at the 3-month follow-up, at which point most participants who maintained food insecurity were classified into the low food insecurity subgroup. Regarding quality of life, the intervention had no impact on EQ-5D-3L, but significantly improved the FACIT-F scores. Regarding physical function, there was a significant improvement in HAQ, EMS, and regularity of exercise practice. No differences were found in hand grip strength after the intervention. Regarding nutritional status, adherence to MD did not change.

**Table 3:** Effects of the intervention program on food insecurity, quality of life, physical function, and nutritional status (N=31)^a^.

Characteristics					Baseline			3-month follow-up			Difference			*P* value
Food security (n=30), n (%)					0 (0)			12 (40)			12 (40)			.001^b^
**Food insecurity, n (%)**					30 (100)			18 (60)			−12 (40)			N/A^c^
	Low food insecurity			26 (87)			17 (57)			−9 (30)				
	Moderate food insecurity			3 (10)			0 (0)			−3 (10)				
	Severe food insecurity			1 (3)			1 (3)			0 (0)				
**Quality of life, mean (SD)**														
	EQ-5D-3L^d^ (n=23)			0.62 (0.29)			0.65 (0.30)			0.03 (0.26)			.58	
	FACIT-F^e^ (n=31)			38.52 (9.32)			41.96 (10.00)			3.82 (8.27)			.02^b^	
**Physical function**														
	HAQ^f^ (n=23), mean (SD)			0.77 (0.70)			0.55 (0.60)			−0.22 (0.38)			.01^b^	
	EMS^g^ (n=10), mean (SD)			19.70 (0.67)			18.20 (1.23)			−1.50 (1.08)			.01^b^	
	Hand grip strength (n=10), mean (SD)			29.35 (7.52)			30.87 (10.44)			1.52 (3.72)			.21	
	**Regular exercise** **(n=31), n (%)**													
		Yes	10 (32)			18 (58)			8 (26)			.02^b^		
	**Days physically active in the last week (45-minutes duration; n=31), n (%)**													N/A
		None	24 (77)			16 (52)			−8 (26)					
		1-2	3 (10)			8 (26)			5 (16)					
		3-4	1 (3)			4 (13)			3 (10)					
		≥5	3 (10)			3 (10)			0 (0)					
**Nutritional status**														
	**PREDIMED^h^ (n=21), n (%)**													.99
		Low adherence to MD^i^	19 (90)			19 (90)			0 (0)					
		High adherence to MD	2 (10)			2 (10)			0 (0)					

^a^Sample size is not constant due to missing data.

^b^*P*<.05.

^c^N/A: not applicable.

^d^EQ-5D-3L: European quality of life questionnaire with five dimensions and three levels.

^e^FACIT-F: Functional Assessment of Chronic Illness Therapy-Fatigue.

^f^HAQ: Health Assessment Questionnaire.

^g^EMS: Elderly Mobility Scale.

^h^PREDIMED: Prevención con Dieta Mediterránea.

^i^MD: Mediterranean diet.

## Discussion

### Principal Findings

To the best of our knowledge, this is the first study to test an eHealth intervention program for older adults who have food insecurity, which is a vulnerable population that is historically not exposed to health innovation. Before implementing a full-scale trial, we conducted this pilot study to explore the feasibility and acceptability of a multidisciplinary 12-week home-based intervention program focusing on improving dietary and physical activity through an interactive TV app. The results of this pilot study reveal aspects of the intervention that may need modification before moving to a full-scale trial to evaluate its effectiveness. This methodology is suggested whenever complex health interventions are developed and implemented in real-life settings [27]. Overall, the intervention program is feasible and highly acceptable. Our findings also provide insights into the promising effects of eHealth interventions on food insecurity, fatigue, and physical function.

During the recruitment period, 177 older adults meeting the eligibility criteria were identified; however, only 17.5% (31/177) were enrolled in the intervention program. This adherence rate was lower than initially anticipated [26] and can be explained by different reasons. First, our recruitment was restricted to selected primary health care centers during a specific period. Thus, we may have identified only those individuals for whom access to health care was not a problem, which may explain their low interest and motivation to engage in an eHealth intervention program at baseline. In addition, a recent study in a Portuguese context reported that older adults with food insecurity admit to reducing their medical visits and stopping medication for economic reasons [9]. Thus, a potential lack of awareness and low prioritization of managing food insecurity may reflect older adults’ need to prioritize other activities perceived as more important. Thus, it might be important to broaden our recruitment strategy in future trials [46-49]. One excellent indicator for a future study, however, was that no participants were lost to follow-up during the study period. This retention rate contrasts with the dropout rate of 45% at the 3-month follow-up found by Van Doorn-Van Atten et al [47] in another study on the feasibility of an eHealth intervention targeting the nutritional status of vulnerable elderly people. Overall, our results suggest that when elderly individuals agree to participate, their engagement with the study will not be a barrier to trial completion.

A similar reason could explain our results regarding one of the primary study outcomes: the feasibility of the intervention program. Our monitoring protocol showed that all participants used the TV app; however, 32% (10/31) self-reported low use at the 3-month follow-up. Knowing that participant compliance is a common barrier to eHealth interventions [16], we integrated several strategies to monitor use and help participants remember to use the TV app (ie, monitoring the number of times participants accessed the TV app, technological reminders, phone calls, and questionnaires). However, these strategies are insufficient, which may present a challenge for future studies. To improve the monitoring process and thus increase participants’ compliance, an emerging strategy is the use of pedometers or accelerometers, which have the potential to objectively and continuously monitor patients and inform researchers about participants’ compliance with physical activity or exercise programs [20,21]. However, the adoption of this strategy in the present intervention program could complicate an already complex intervention while considering the characteristics of the end users, without guaranteeing that participants will use the monitoring instruments during their exercises or daily routine activities.

The baseline lack of participants’ interest in using the TV app may be another explanation for self-reported low use. Even those participants who self-reported consistent use of the TV app stated that they had low interest in eHealth interventions in the baseline assessment. Nonetheless, after the intervention, we observed a substantial increase in participants’ interest in eHealth interventions, both for improving food insecurity and for other purposes. This important result clearly justifies future investment in health innovation for this population.

In line with our feasibility results, the intervention program was highly accepted by participants both before and after the intervention. Acceptability, a multifaceted construct that reflects the extent to which people receiving a health care intervention consider it to be appropriate based on anticipated or experienced cognitive and emotional responses, was evaluated using constructs suggested by the theoretical framework of acceptability [37]. As expected, some of these constructs showed a significant decrease between the baseline and 3-month follow-up. Our eHealth intervention comprises a highly innovative digital program directed toward a vulnerable older adult population with low educational and socioeconomic status, low digital literacy, and a lack of regular exposure to health innovation, which are well-known barriers to eHealth adoption and implementation [24]. Thus, we anticipated that an innovative digital intervention would initially be met with high levels of acceptability. Later, however, as the intervention program involved active participation and commitment to physical exercise and a healthy diet, a decline in acceptability was observed. Despite this decrease, the median values of all acceptability constructs remained very high after the intervention.

Comparison of the present results with those of previous studies on the feasibility and acceptability of eHealth interventions focused on nutrition and physical activity among vulnerable older adults is challenging. The small number of previous studies and the high degree of heterogeneity in their designs, samples, interventions, and methods makes any comparison complex. Even so, our feasibility and acceptability results appear to be superior to those reported by Kraft et al [49] and Van Doorn-Van Atten et al [47].

Although impact analysis was not the primary objective of this study, the observed changes in key clinical outcomes revealed very promising short-term effects that support future randomized controlled trials. To the best of our knowledge, this is the first behavior change eHealth intervention that appears to effectively reduce food insecurity status and severity in a sample of older adults. The intervention program also showed improved fatigue and physical function of the participants, including their functional status, performance and mobility, and regularity of exercise. These results are in line with previous systematic reviews showing the effectiveness of eHealth interventions in improving the physical activity and physical functioning of older populations [20,21]. However, despite the systematic review by Marx et al [19] showing that eHealth malnutrition–related interventions improve the HRQoL and nutritional status of older adults, our intervention had no effect on these outcomes. These results could be explained by factors such as older age and greater physical, social, and economic vulnerabilities of our sample combined with the short follow-up period and use of a single instrument to evaluate the nutritional status. Longer follow-up periods and additional instruments for evaluating nutritional status (eg, 24-hour dietary recalls) should be considered for future effectiveness analysis.

### Strengths and Limitations

This study has several limitations that need to be addressed in future full-scale trials. First, the low adherence of older adults may reflect selection bias. In addition to the large number of individuals who did not agree to participate in the study, 21 individuals were excluded because of technological problems with the installation of the TV app, which is an important limitation that needs to be addressed in future studies. Second, our feasibility and acceptability analyses were based exclusively on quantitative data collected through a structured questionnaire. The collection of qualitative data using semistructured interviews or focus groups could provide more detailed insights into participants’ perspectives and thus reveal additional important aspects of the intervention in need of modification. Third, our short follow-up period prevented the analysis of the long-term effects of the intervention program. Fourth, the absence of a control group and the nonrandomized design of our study did not allow us to analyze the causal effects of the intervention program or to explore important feasibility aspects in preparation for a future randomized clinical trial, such as the randomization process and blinded outcome assessment. Finally, our pilot study involved a high proportion of missing data for almost all outcomes, which may compromise some of the data analysis performed.

Despite these limitations, this study’s results can inform potential strategies for mitigating a growing global public health problem, which is expected to reach historic levels because of the COVID-19 pandemic [5,6]. Moreover, this pandemic is expected to cause an unprecedented social and economic crisis with devastating consequences for the most vulnerable populations [5,6]. Thus, there is an urgent need to develop feasible, acceptable, and effective interventions to promote healthy lifestyles and enhance well-being and quality of life among older adults. Our study represents the first step toward designing an effective intervention. Its strengths include the real-life health care context from which participants were recruited and the use of a validated instrument from the USDA Household Food Security Survey Module to identify eligible participants [29], which increases the external validity of our study. In addition, the study design was based on international frameworks for the design and implementation of complex and innovative interventions [27,28] and the assessment of acceptability constructs [37]. The strengths of this eHealth intervention program include (1) the early involvement of a multidisciplinary team and TV app end users in the development of the intervention program; (2) a multicomponent program aimed at improving several health outcomes (ie, nutritional, physical, and quality of life) based on behavioral change techniques; (3) use of a familiar ICT in participants’ daily lives; and (4) close monitoring by the research team during the intervention period.

### Conclusions

In conclusion, the findings of this pilot study reveal that our multidisciplinary 12-week home-based eHealth intervention program is a feasible and highly accepted method for improving the dietary and physical activity of older adults using a TV app, thus supporting a future full-scale trial. This intervention program not only reduced the proportion of older adults with food insecurity but also improved their fatigue and physical function.

## Abbreviations

EMS: Elderly Mobility Scale
EQ-5D-3L: European Quality of Life Questionnaire with five dimensions and three levels
FACIT-F: Functional Assessment of Chronic Illness Therapy-Fatigue
HAQ: Health Assessment Questionnaire
HRQoL: health-related quality of life
ICT: information and communication technology
MD: Mediterranean diet
PREDIMED: Prevención con Dieta Mediterránea
TV: television
USDA: United States Department of Agriculture
